# Alcohol Use and Abuse Among Family Caregivers of People Living with Dementia in the United States: A Scoping Review

**DOI:** 10.3390/ijerph21111525

**Published:** 2024-11-17

**Authors:** Afeez A. Hazzan, Jessica L. Sniatecki, Gary Metz, Jamia Williams

**Affiliations:** 1 Department of Healthcare Studies, State University of New York, Brockport, NY 14420, USA; jsniatecki@brockport.edu (J.L.S.); gmetz@brockport.edu (G.M.); 2 Spencer S. Eccles Health Sciences Library, The University of Utah, Salt Lake City, UT 84112, USA; jamia.williams@utah.edu

**Keywords:** aging, dementia, family caregivers, alcohol use, alcohol abuse, scoping review

## Abstract

The population of the United States is rapidly aging due to a number of factors, such as lower fertility rates and increases in life expectancy. Globally, dementia is a leading cause of disability among older adults, affecting approximately 50 million people. Family caregivers, who often have to sacrifice their health and well-being, provide most of the care needed by older adults living with dementia. Further, alcohol is one of the most commonly used substances in the United States. Considering the stress and unfavorable health outcomes that are associated with caring for a loved one with dementia, it is important to understand the prevalence of alcohol use and abuse among family caregivers. In this study, Arksey and O’Malley’s five-stage approach for a scoping review is used to examine the nature and scope of a body of research related to alcohol use and abuse among family caregivers of people living with dementia in the United States. Overall, the results show a paucity of research on the prevalence and implications of alcohol use among family caregivers. The identified studies suggest that family caregivers may be less likely to misuse alcohol than non-caregivers. However, additional population-wide studies are needed.

## 1. Introduction

### 1.1. Alcohol Use in the United States

Alcohol is one of the most commonly used substances in the United States. According to the 2019 National Survey on Drug Use and Health, 86.7% of adults aged 26 and older reported lifetime alcohol use, 69.1% reported use in the last year, and 55% reported use in the last month [1]. Binge drinking was also indicated for 24.5% of this group, with 6% reporting heavy alcohol use [1]. 

Further, the 2019 National Survey on Drug Use and Health found that almost 15 million people aged twelve or older in the United States meet the criteria for an alcohol use disorder [2]. Of these, only 7.2% had been involved in treatment in the past year, indicating the need for more access to treatment. Importantly, alcohol use causes a significant health burden in the United States and is a leading cause of preventable death, with an average of approximately 95,000 annual deaths and 2.7 million years of potential life lost attributed to alcohol use between 2011 and 2015 [3]. 

In addition, studies have shown that alcohol use and alcohol-related deaths increased significantly during the recent COVID-19 pandemic. A study comparing the number of alcohol-related deaths in 2019 and 2020 found that these deaths increased by 25% between the two years [4]. Considering that all the other causes of mortality increased by 16% during the period under study, alcohol-related deaths increased at a higher rate [4].

### 1.2. Dementia

Dementia is a leading cause of disability among older adults, affecting approximately 50 million people around the world [5]. Alzheimer’s disease is the most common form of dementia, comprising 60–70% of identified cases [5]. Dementia often causes significant declines in cognitive functioning in affected individuals as well as significant strains on caregivers and families [5]. Importantly, there is currently no cure for dementia, and there are no treatments available to stop its progression [5,6,7]. Though dementia is much more common among adults aged 65 and older, approximately 9% of cases are early-onset dementia [5,6]. These types of dementia are characterized by progressive brain deterioration, memory loss and an inability to independently care for oneself at a relatively young age, usually age 65 [6]. 

In the United States, it is estimated that in 2021, one in nine adults aged 65 and older was living with Alzheimer’s dementia, or 6.2 million people [7]. It is anticipated that these numbers will grow substantially in the coming years as the population of older adults continues to increase [7].

### 1.3. Dementia Caregivers, Stress, and Health

As dementia symptoms progress, individuals with these conditions often require care to manage their symptoms and live independently. Family caregivers provide a substantial amount of the care needed by older adults living with dementia [8]. These caregivers perform important duties such as dressing, meal preparation, care planning, and financial management [7,8]. According to the Alzheimer’s Association, “more than 11 million Americans provide unpaid care for people with Alzheimer’s or other dementias” [7]. Unpaid caregiving is very common in the United States, comprising 83% of the care for older adults. Almost half of these caregivers provide care to someone living with dementia [7]. Approximately 30% of these caregivers are older adults themselves [7].

Caregiving for a person with dementia is significantly correlated with stress. Compared with family caregivers of people without dementia, studies have shown that twice as many caregivers of people with dementia have substantial emotional, financial and physical difficulties [7]. Several studies have found that providing care for someone with dementia is associated with psychological difficulty, such as depression and anxiety [9,10,11,12,13,14,15,16,17,18,19]. In addition, studies have found significantly higher levels of psychological distress and stress among family caregivers of people with dementia compared to other caregivers and non-caregivers [9]. Additionally, family caregivers of people with dementia have lower levels of self-efficacy, subjective well-being, and physical health compared to other caregivers and non-caregivers [9]. Further, a two-year longitudinal study involving Alzheimer’s caregivers found substantial evidence of high levels of depressive symptoms among both male and female participants [18]. Similarly, Mahoney et al. (2015) found high rates of depression and anxiety symptomology among family caregivers of individuals with Alzheimer’s disease [16]. A study in Spain comparing dementia caregivers to non-caregivers found that “reported mental health (mental, emotional role, and social categories of SF-36) of the carers was 22% lower than that of the non-caregivers, but both groups were similar in physical health” [20]. 

Several studies have suggested that dementia caregivers are more likely to experience health issues and negative health behaviors [13,14,19,20,21,22]. Research has demonstrated that compared with non-caregivers, dementia caregivers are at increased risk of coronary heart disease [23] and respiratory system symptoms [13], and they are more likely to experience sleep disturbance [24]. In a study involving spousal dementia caregivers, Connell (1994) found that 40% reported negative impacts on their physical health, including arthritis, high blood pressures, and back problems [25].

Research has also explored whether dementia caregivers are more likely to use substances compared to non-caregivers. McKibbin et al. (1999) found that dementia caregivers who were wives and mothers used alcohol more frequently than their peers, though the quantities of use were similar [26]. In a recent study, Hernandez Chilatra et al. (2024) found that 18.1% of family caregivers of people living with Alzheimer’s disease and related dementia screened as positive for hazardous drinking, as measured by the Alcohol Use Disorders Identification Test-Consumption [21]. Substance use may be a coping mechanism for dementia caregivers and may lead to increased rates of use [25]. Further, Connell (1994) found that approximately 1/3 (34.1%) of spousal caregivers engaged in alcohol use and/or use of medications as a means of coping with caregiver stress [25].

The purpose of the current study was to conduct a scoping review to examine the issues of alcohol addiction and abuse among family caregivers of people living with dementia. In addition, this study examined interventions for addressing these issues. 

## 2. Materials and Methods

This scoping review has been registered with the Open Science Framework (OSF). The OSF registration DOI is https://doi.org/10.17605/OSF.IO/9KUH5 (Accessed on 30 October 2024). 

### 2.1. Study Objectives

The objectives were to (a) determine the rate or prevalence of alcohol use and abuse among family caregivers of people living with dementia in a community setting in the United States; (b) summarize the perceived reasons for alcohol use and abuse among family caregivers of people living with dementia in these settings; and (c) describe the impact of the recent COVID-19 pandemic on alcohol use and abuse among members of this population.

### 2.2. Guiding Framework

A scoping review was determined to be appropriate for this study because it makes it possible to examine the nature and scope of a body of research related to alcohol use and abuse among family caregivers of people living with dementia in the United States, identify gaps in the literature covering these topics, and guide decisions about whether to proceed with alternate research methodologies or approaches for examining this research topic [27,28,29]. Specifically, Arksey and O’Malley (2005)’s five-stage approach for scoping reviews was followed in this study [27]. The different stages involved in this approach, as well as detailed explanations of the steps involved, are explained below. 

### 2.3. Stage 1: Identifying the Research Questions

Stage 1 of Arksey and O’Malley’s five-stage approach for scoping reviews begins with identification of the research questions the study intends to address. The research questions for the current scoping review were guided by the Population, Intervention, Context, Outcome (PICO) approach [30]. This approach is depicted in Table 1. The specific research questions examined in this scoping review are as follows.

1. What is the rate/prevalence of alcohol use and abuse among family caregivers of people living with dementia in a community setting?
2. What are the perceived reasons for alcohol use and abuse among family caregivers of people living with dementia in a community setting?
3. How has the COVID-19 pandemic affected alcohol use and abuse among family caregivers of people living with dementia in a community setting?

### 2.4. Stage 2: Identifying Relevant Studies

The search strategy for this scoping review study was developed and implemented by the research team in collaboration with a health sciences librarian. This involved searching peer-reviewed studies available in PubMed, CINAHL Complete, as well as PsycINFO. Further, the search strategy was adapted for each database used (see Appendix A for the PubMed search strategy used for this study). Using the search strategy, we obtained studies focusing on issues related to alcohol use and abuse among family caregivers of people living with dementia in a community setting in the United States. 

Specifically, we were interested in studies that described the rate/prevalence of alcohol use and/or abuse among family caregivers of people living with dementia in a community setting, as well as the perceived reasons or justifications for this behavior. In addition, we were interested in estimating the impact of the COVID-19 pandemic on the rate/prevalence of alcohol use and abuse among members of this population. 

Further, all study designs (i.e., quantitative, qualitative, mixed-methods, etc.) were eligible for inclusion in this review. However, studies had to be conducted in the United States, available in the English language, and published within the last 20 years. 

Citations from each database searched were uploaded to a web-based program. This allowed the researchers to identify and remove duplicates [31]. Also, the title and abstract of each identified article were screened. To identify possible additional studies, the reference lists of the included articles were hand-searched. Finally, gray literature was identified by searching the internet and asking experts in the field to help recommend other relevant publications for inclusion in the study.

**Figure 1:**
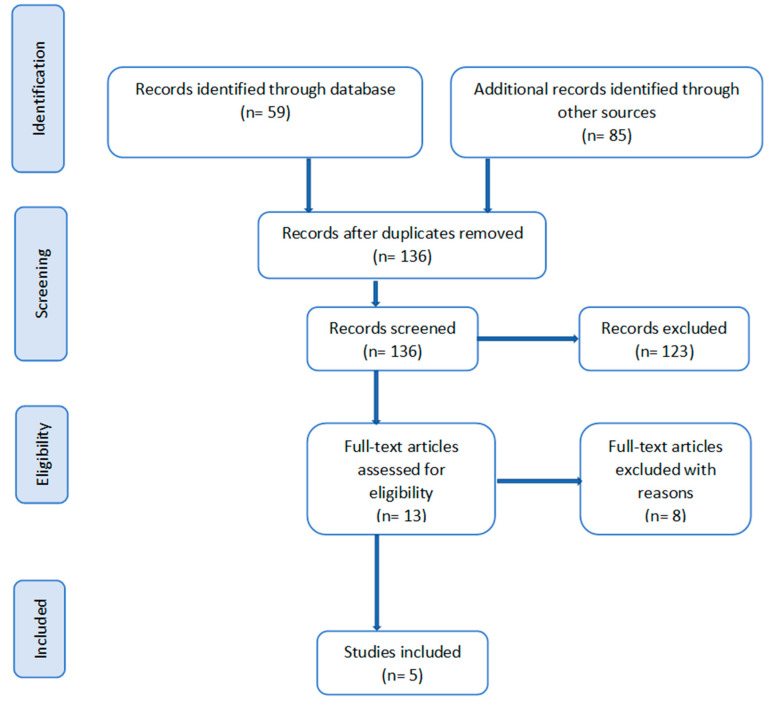
PRISMA flow diagram for the study.

Figure 1 below shows the PRISMA flow diagram of the charting process for this study [32,33]. 

### 2.5. Stage 3: Article Selection

Once the relevant studies were identified, screening of the articles was independently conducted by two researchers. Any disagreements in terms of the article selection were discussed until a consensus was reached on the exclusion status of the article in question. Appendix B provides sample screening criteria for this scoping review.

The key inclusion criteria were as follows.

- Published between 2003 and July 2023
- Studies conducted in the United States
- Studies written in the English language
- Focused on family caregivers of people living with any type of dementia in a community setting
- Studies must address issues related to alcohol addiction and/or abuse among these family caregivers

### 2.6. Stage 4: Data Charting

From the initial 136 unique records that were identified in this scoping review, five studies were included. The key study characteristics were extracted from the five studies that made it to the full-text screening stage (see Table 2). These characteristics included demographic profiles of the family caregivers and care recipients included in each study, as well as the study context. Other characteristics that were extracted from these studies included how alcohol use or abuse was measured, the reported rate, prevalence, or observation regarding alcohol use and abuse, as well as the reported reasons or justifications for alcohol use or abuse.

Table 3 summarizes the pertinent information extracted from each included study.

## 3. Results

### 3.1. Stage 5: Collating, Summarizing, and Reporting the Study Results

The final stage of a scoping review process using Arksey and O’Malley’s (2005) five-stage approach involves the collating, summarizing, and reporting of the results extracted from the included studies [27]. All five studies included in the final analysis met the full criteria for this study, including the publication date, being conducted in the United States, and being published in the English language. Further, the studies focused on family caregivers who are providing care to a community-dwelling person living with dementia. In addition, the included studies addressed issues related to alcohol use or abuse among the family caregivers. 

### 3.2. Measuring Alcohol Use and Abuse Among Family Caregivers of People Living with Dementia in a Community Setting

The included studies reported alcohol use or abuse among family caregivers of people living with dementia. The data extraction included how alcohol use or abuse was measured in each study, as well as the reported rate, prevalence, or observations regarding alcohol use and abuse. Several approaches were used to measure alcohol use or abuse in these studies, including dietary guidelines [34], the Health Behaviors Inventory [36], as well as the Michigan Alcohol Screening Test [37]. Further, one of the studies measured alcohol use by scoring it on an ordinal scale based on the number of days on which caregivers had at least one alcoholic drink in the past month, with scores ranging from “0 days” to all “30 days” [38]. Finally, another study assessed alcohol use during the past month with three measures, including (1) binge drinking, (2) average drinks per week, and (3) heavy drinking [35]. 

### 3.3. Reported Rate, Prevalence, or Observation Regarding Alcohol Use or Abuse 

The studies included in this scoping review reported data regarding the rate, prevalence, or the author’s observation regarding alcohol use or abuse. For example, Gottschalk et al. (2020) found that caregiving was associated with more responsible alcohol consumption (OR = 0.86, 95% CI [0.81–0.92]) [34]. In addition, Shifren and Chong (2012) reported that caregivers and non-caregivers differed significantly in terms of alcohol consumption (i.e., 5.67(1.11) vs. 5.01(1.10), t-value = 2.66) [36]. The authors found that former young caregivers report drinking less alcohol than non-caregivers.

On the other hand, Secinti et al. (2022) found that binge drinking did not differ between caregiver groups and non-caregivers [35]. However, caregivers of patients with dementia were less likely to report heavy drinking than non-caregivers (OR = 0.73, *p* < 0.05). According to VandeWeerd et al. (2013), caregivers with alcohol problems were three times as likely to be violent with the elders for whom they were providing care (*p* = 0.041; OR = 3.217; CI = 2.382–4.775) [37]. The authors also reported that alcoholism on the part of caregivers (*p* = 0.052; OR = 6.176; CI = 4.511–9.706) was significantly related to the likelihood of physical mistreatment [37]. Further, von Kanel et al. (2019) found that greater care recipient dementia was associated with less alcohol consumption (r = −0.20, *p* = 0.022) [38]. In addition, greater care recipient functional impairment was associated with less alcohol consumption (r = −0.26, *p* = 0.002). 

### 3.4. Reported Reasons or Justifications for Alcohol Use or Abuse Among Family Caregivers

In elucidating the negative association between caregiving and alcohol consumption, Gottschalk et al. (2020) explained that alcohol consumption entails more and longer-lasting consequences related to the inability to manage caregiving responsibilities [34]. For Secinti et al. (2022), the lower likelihood of heavy drinking among family caregivers may be related to the caregiver’s older age, the need for continual surveillance of patient activities, and healthier relatives assuming caregiving responsibilities [35]. 

Further, Shifren and Chong (2012) explained that the lower likelihood of family caregivers consuming alcohol could be due to the fewer opportunities they have to socialize [36]. The authors also explained that the former young caregivers enrolled in the study may have experienced life with someone who was an alcoholic and therefore could be more reluctant to engage in risky health behaviors such as alcohol consumption [36].

In describing the impact of alcohol abuse among family caregivers, VandeWeerd et al. (2013) explained that alcohol abuse in caregivers increases the likelihood of physical mistreatment of care recipients [37]. Finally, in reporting their finding that greater care recipient functional impairment was associated with less alcohol consumption, von Kanel et al. (2019) explained that poor/fair self-rated health (SRH) versus at least good SRH reflects an inclusive measure of low health as well as caregiving-specific stress in dementia caregivers [38].

Notably, only two of the included studies were published after COVID-19 was declared a pandemic. These studies are Gottschalk et al. (2020) and Secinti et al. (2022) [34,35]. However, neither of these studies mentioned the possible impact of the COVID-19 pandemic on alcohol use and abuse among family caregivers. These findings are not surprising because the data used in these studies were not collected during the COVID-19 pandemic. Interestingly, a recent study included data collected before and during COVID-19 but did not discuss the impacts of the pandemic [21]. 

## 4. Discussion

The results of this scoping review highlight the paucity of research focused on the prevalence and implications of alcohol use among family caregivers of older adults with dementia. Only five studies were identified, and these used a variety of methodological approaches to assess alcohol use/misuse among family caregivers of older adults living with dementia, making it challenging to draw universal conclusions about this topic. The lack of comprehensive research in this area hampers our ability to understand the true status of this issue among caregivers of older adults with dementia.

The existing studies suggest that caregivers may be less likely to misuse alcohol than non-caregivers [34,36]; however, additional studies are needed to confirm this pattern. Similarly, binge drinking has been associated with several negative outcomes, including elder abuse [37] and inability to provide appropriate care for a loved one with dementia [34]. Although this was not directly investigated in the included studies, it is possible that some family caregivers may be intentionally refraining from these behaviors in order to optimize the level or quality of care provided to their loved ones. Indeed, the family caregiver’s own quality of life has been shown to be an important factor for the quality of care provided to older adults living with dementia [28,39,40].

In addition to the findings presented above, we conducted a search of the literature examining the issue of alcohol use and abuse among family caregivers of people living with dementia prior to the earliest eligible year of 2003. However, we did not find any studies that suggest major changes in the drinking patterns among caregivers of individuals living with dementia as described in this study. However, more recent studies suggest that alcohol abuse/misuse among family caregivers may be related to the caregiver burden or stress [41,42]. Similarly, evidence suggests that the issue of alcohol use/abuse among family caregivers of individuals living with other chronic illnesses and disabilities may also be related to a high level of caregiver burden or stress [42]. 

Clearly, more studies are needed to fully understand the impact of alcohol use/misuse on caregiving behaviors and responsibilities. Such research could provide important insights into how to best assess this behavior among caregivers of individuals with dementia and identify those most at risk of problematic drinking, as the existing research found variation based on the dementia symptomatology and the functional impairment level of the family member receiving care [38]. Additional data could also help identify resources and supports for this populations to help minimize the negative effects for those for whom they provide care. 

Moreover, additional research is needed on the impact of the COVID-19 pandemic on alcohol use among caregivers. No studies were found that examined this issue among caregivers of individuals with dementia, though changes in rates of alcohol consumption during the pandemic have been well documented in various populations within the United States [43]. Further, interventions that are targeted toward improving the quality of life of family caregivers may ameliorate risky behaviors such as alcohol use and abuse. Such interventions have been successfully implemented for similar caregiver populations with promising results [44,45].

## 5. Conclusions

Although there is a lack of robust research examining the issue of alcohol use and abuse among family caregivers of older adults living with dementia, the findings from this study show that family caregivers are less likely to engage in this behavior. The dearth of research on this topic means that additional research needs to be conducted to confirm these findings as well as to gain a wider perspective on the topic. A population-wide longitudinal study examining alcohol use and abuse among family caregivers of older adults living with dementia will be in order. Finally, interventions that are targeted toward improving the behavioral health, including mental health and substance use disorders, among family caregivers of older adults with dementia will be in order.

## Figures and Tables

**Table 1:** PICO analysis for the development of the scoping review research questions.

PICO		Headings Description
P	Population/participants	Unpaid caregivers of people living with dementia
I	Intervention	Interventions or issues related to alcohol use and abuse among family caregivers of people living with dementia
C	Context	Family caregivers of people living with dementia in a community setting; studies conducted in the United States; all study designs eligible; studies published within the last 20 years; available in the English language
O	Outcome	Alcohol use, alcohol addiction, alcohol abuse (e.g., heavy drinking, binge drinking)

**Table 2:** Characteristics of the family caregivers and patients, and aims/objectives of the included studies.

Study (Authors and Year)	Characteristics of the Family Caregiver	Characteristics of the Person with Dementia	Study Context/Concept	Study Method	Study Aims/Objectives
Gottschalk et al. (2020) [34]	Caregiver status (yes/no). This was derived from the question “During the past 30 days, did you provide regular care or assistance to a friend or family member who has a health problem or disability?”	Non-caregivers (NCGs) vs. dementia caregivers (DCGs)	Utilized data from the 2017 wave of the Behavioral Risk Factor Surveillance System (BRFSS) of the Center for Disease Control. These include data from US residents in 50 states, the District of Columbia as well as three US territories.	Cross-sectional study	Aim #1: To address the hypothesis that worse physical health in CGs is attributable to health behavior by comparing NCGs to CGs and to DCGs regarding selected behavioral risk factors (alcohol consumption, smoking behavior, overweight/obesity, insufficient physical activity). Aim #2: To assess the relationship between caregiving-related variables (caregiving intensity, length of caregiving, relationship to care recipient, and caregiving tasks) and behavioral risk factors within the group of CGs and DCGs.
Secinti et al. (2022) [35]	Caregiving status was categorized based on the following questions: “During the past 30 days, did you provide regular care or assistance to a friend or family member who has a health problem or disability?” and “What is the main health problem, long-term illness, or disability that the person you care for has?”	People with dementia	BRFSS data from the five most recent cross-sectional waves were combined: 2015 (24 states), 2016 (21 states and territories), 2017 (12 states), 2018 (5 states), and 2019 (10 states).	Pooled cross-sectional	Aim #1: To compare health behavior between the caregiver groups and non-caregivers using hierarchical regression analyses with two steps. Aim #2: To examine the relationships between caregiving intensity and each health behavior in the caregiver samples using hierarchical binary logistic or Poisson regression with three steps.
Shifren and Chong, (2012) [36]	Early caregiving, defined as the provision of basic activities of daily living (ADLs) (i.e., bathing, dressing, feeding) and/or instrumental activities of daily living (IADLs) (i.e., doing finances, grocery shopping, providing transportation, household chores) for a parent or adult relative while the caregiver was under 21 years old. In addition to the ADLs, IADLs, and the age requirements, individuals had to provide care for at least several hours a day for a 1 month period to be considered a caregiver.	Older adults with Alzheimer’s disease	Part of ongoing research on former young caregivers that began in 1998.	Cross-sectional study	Aim #1: To provide descriptive information on these former young caregivers’ adult health-related behaviors. Aim #2: Comparing former young caregivers’ health-related behaviors to non-caregiver samples. Aim #3: Assessing the relationship between former young caregivers’ health-related behaviors and their mental health.
VandeWeerd et al. (2013) [37]	Caregivers for older adults diagnosed with AD according to the NINCDS/ADRDA criteria. Also includes dementia of the Alzheimer’s type according to the DSM-III-R or DSM-IV in the three years leading to their enrollment in the study.	Specifically older adults who have been diagnosed with AD and receive treatment from memory disorder clinics/ members of Alzheimer’s Associations during the enrollment period.	Older adults with Alzheimer’s disease and dwelling in the community in state of Florida. Must also be using the risk and vulnerability model of elder mistreatment.	Analysis of secondary data collected from the aggression and violence in community-based Alzheimer’s families grant.	Examines risk factors for physical mistreatment among community-dwelling older adults with AD in the state of Florida utilizing the risk and vulnerability model of elder mistreatment.
von Kanel et al. (2019) [38]	Caregivers enrolled between 2/2015 and 11/2017 in the University of California, San Diego (UCSD) Alzheimer’s Caregiver Study for an RCT.	Spouse with dementia	Examined 134 elderly (≥55 years) caring for a spouse with dementia and rated their own health (SRH).	Cross-sectional study	Sought to identify demographic factors (for example, age, sex, education), health characteristics (for example, health behaviors, physical health indicators, psychosocial factors) and caregiving-specific stressors (for example, years of caregiving, dementia severity, functional impairment of the care recipient, perceived caregiver burden) that may differentiate dementia caregivers with poor/fair SRH from dementia caregivers with good, very good, or excellent SRH.

**Table 3:** Summary of issues regarding alcohol use and abuse among family caregivers.

Study (Authors and Year)	How Alcohol Use or Abuse Was Measured	Reported Rate, Prevalence, or Observation Regarding Alcohol Use or Abuse	Reported Reasons/Justification
Gottschalk et al. (2020) [34]	2015–2020 Dietary Guidelines for Americans	Caregiving was associated with more responsible alcohol use (OR = 0.86, 95% CI [0.81–0.92]).	Negative association between caregiving and alcohol consumption. Reasons include alcohol consumption entailing longer-lasting consequences related to the inability to manage caregiving duties.
Secinti et al. (2022) [35]	Assessed alcohol use during the past month with 3 measures: (1) binge drinking (i.e., male caregivers reporting having ≥5 drinks or female caregivers reporting having ≥4 drinks on ≥1 occasion in the past month; yes versus no), (2) average drinks per week (continuous), and (3) heavy drinking (i.e., male caregivers reporting having >14 drinks per week or female caregivers reporting having >7 drinks per week; yes versus no)	Binge drinking did not vary between caregivers and non-caregivers; only caregivers of patients with dementia were less likely to report heavy drinking than non-caregivers (OR = 0.73, *p* < 0.05).	Lower likelihood of heavy drinking related to caregiver’s older age, need for continual surveillance of patient activities, as well as healthier relatives assuming caregiving responsibilities.
Shifren and Chong (2012) [36]	The Health Behaviors Inventory (HBI). The HBI contains ten items, including drinking.	Caregivers and non-caregivers differed significantly on alcohol consumption {i.e., 5.67(1.11) vs. 5.01(1.10), t-value = 2.66 (*p* = 0.009). Specifically, former young caregivers report drinking less alcohol than non-caregivers.	Caregivers may not have as much opportunity to socialize with others. Also, the former young caregivers enrolled in the study may have life experiences with someone who was an alcoholic. Therefore, caregiving for a parent or adult relative with alcoholism could reduce a caregiver’s desire for alcohol.
VandeWeerd et al. (2013) [37]	Michigan Alcohol Screening Test (MAST)	Caregivers with alcohol problems were three times as likely to be violent with elders for whom they were providing care (*p* = 0.041; OR = 3.217; CI = 2.382–4.775). Alcoholism on the part of caregivers (*p* = 0.052; OR = 6.176; CI = 4.511–9.706) was significantly related to the likelihood of physical mistreatment.	Alcohol abuse in caregivers increase the likelihood of physical mistreatment.
von Kanel et al. (2019) [38]	This was scored on an ordinal scale based on the number of days on which caregivers had at least one alcoholic drink in the past month (scores ranges from 0 “0 days” to 6 = “all 30 days”).	Alcohol consumption (all caregivers, *n* = 134), score, mean (SD) 0 to 6 2.03 (2.26) Greater care recipient dementia was related with less alcohol consumption (r = −0.20, *p* = 0.022). Greater care recipient functional impairment was associated with less alcohol consumption (r = −0.26, *p* = 0.002).	Poor/fair self-rated health (SRH) vs. at least good SRH indicates an inclusive measure of low health and caregiving-specific stress in dementia caregivers.

## Data Availability

The data presented in this study are available on request from the corresponding author.

## Appendix A. Sample PubMed Search Strategy

((“Caregivers”[Mesh] OR “Carer*”OR “Care Giv*” OR “Caregiv*”) AND (“Dementia”[Mesh] OR “Frontotemporal Dementia”[Mesh] OR Dementia OR Alzheimers disease OR “vascular dementia” OR “dementia with lewy bodies” OR “frontotemporal lobar dementia” OR “young onset dementia” OR “ mixed dementia” OR “parkinson’s dementia”)) AND (“Alcoholism”[Mesh] OR “Alcohol Dependence” OR “Alcohol Use Disorder” OR “Alcohol addiction” OR “alcohol use” OR “alcohol abuse” OR “alcohol intoxication” OR “alcohol poisoning” OR “heavy drinking” OR “binge drinking”)

## Appendix B. Sample Screening Criteria

1. Is this a peer-reviewed study (i.e., non-peer-reviewed studies or conference abstracts should be excluded)?
2. Does the study population consist of unpaid family caregivers of people with Alzheimer’s disease or other types of dementia?
3. Did the study (i.e., full article) describe or report any issues related to caregiver or unpaid caregiver or family caregiver or informal caregiver?
4. Did the study report any quantitative or qualitative data?
5. Did the study report data for any alcohol-related topic among caregivers (please recognize any related description of this issue)?
